# Characterization of the De Novo Biosynthetic Enzyme of Platelet Activating Factor, DDT-Insensitive Cholinephosphotransferase, of Human Mesangial Cells

**DOI:** 10.1155/2007/27683

**Published:** 2007-06-05

**Authors:** Alexandros Basilios Tsoupras, Elizabeth Fragopoulou, Tzortzis Nomikos, Christos Iatrou, Smaragdi Antonopoulou, Constantinos Alexandros Demopoulos

**Affiliations:** ^1^Faculty of Chemistry, School of Sciences, National and Kapodistrian University of Athens, Panepistimioupolis, 15771 Athens, Greece; ^2^Department of Science of Dietetics and Nutrition, Harokopio University, 70 El. Venizelou Street, 176 71 Athens, Greece; ^3^Centre for Nephrology, G. Papadakis General Hospital of Nikea-Pireaus, 3 Mandouvalou Street, 18454 Athens, Greece

## Abstract

Platelet activating factor (PAF), a potent inflammatory mediator, is implicated in several proinflammatory/inflammatory diseases such as glomerulonephritis, glomerulosclerosis, atherosclerosis, cancer, allergy, and diabetes. PAF can be produced by several renal cells under appropriate stimuli and it is thought to be implicated in renal diseases. The aim of this study is the characterization of DTT-insensitive cholinephosphotransferase (PAF-CPT) of human mesangial cell (HMC), the main regulatory enzyme of PAF *de novo* biosynthetic pathway. Microsomal fractions of mesangial cells were isolated and enzymatic activity and kinetic parameters were determined by TLC and in vitro biological test in rabbit washed platelets. The effect of bovine serum albumin (BSA), dithiothreitol (DTT), divalent cations (Mg^2+^ and Ca^2+^), EDTA, and various chemicals on the activity of PAF-CPT of HMC was also studied. Moreover, preliminary in vitro tests have been performed with several anti-inflammatory factors such as drugs (simvastatin, IFNa, rupatadine, tinzaparin, and salicylic acid) and bioactive compounds of Mediterranean diet (resveratrol and lipids of olive oil, olive pomace, sea bass “*Dicentrarchus* labrax,” and gilthead sea bream “*Sparus aurata*”). The results indicated that the above compounds can influence PAF-CPT activity of HMC.

## 1. INTRODUCTION

Platelet activating factor (PAF, 1-*O*-alkyl-2-acetyl-*sn*-glycero-3-phosphocholine) [1–3], a potent phospholipid mediator with a wide variety of biological activities, is involved in a number of proinflammatory/inflammatory manifestations, such as glomerulonephritis, glomerulosclerosis, atherosclerosis, cancer, allergy, and diabetes.

Many different cells, including leukocytes, platelets, macrophages, neutrophils, lymphocytes, endothelial cells, and renal cells can produce PAF under appropriate stimuli [4]. Two enzymatic pathways of PAF biosynthesis have been described [5]. The *de novo* pathway entails a specific stepwise sequence of reactions ending with a transfer of the phosphocholine base group from CDP-choline to 1-*O*-alkyl-2-acetyl-*sn*-glycerol (AAG) by a dithiothreitol- (DTT-) insensitive cholinephosphotransferase (PAF-CPT), while in the *remodeling* pathway the final step includes an acetylation of l-alkyl-2-lyso-*sn*-glycero-3-phosphocholine (Lyso-PAF) by a specific acetyltransferase (Lyso-PAF-AT). On the other hand, the main enzyme for PAF degradation is PAF-acetylhydrolase (PAF-AH) [6].

In general, it has been proposed that the *de novo* pathway should mainly contribute to PAF synthesis for maintaining its basal levels under physiological conditions, whereas the *remodeling* pathway should be more involved in the production of PAF during inflammatory responses [7, 8]. However, the information collected so far concerning PAF biosynthetic pathways suggest that the contribution of the aforementioned enzymes to PAF synthesis depends on several factors under physiological and pathological conditions [8–13], and so the above point of view should be reevaluated and further studied.

The important regulatory enzyme of the *de novo* route, PAF-CPT, is widely distributed among mammalian tissues and is located on the cytoplasmic surface of the endoplasmic reticulum [8]. It has been found in a variety of rat tissues [7, 8, 12, 14] with the spleen, lung, liver, and kidney exhibiting the highest activities. Human renal cell carcinoma [13], porcine spleen [11], as well as human neutrophils, human cerebrum, fetal rabbit lungs, and unfertilized mouse oocytes, zygotes, and preimplantation embryos [8, 15] also contain significant amounts of PAF-CPT.

PAF-CPT has been solubilized from porcine spleen microsomes using digitonin [11]. Although the activity of the solubilized enzyme was relatively stable, further purification by sequential chromatography caused a remarkable decrease in enzyme activity, which was partially recovered by the exogenously addition of phospholipids such as egg phosphatidylcholine, and so forth. [11]. In contrast, dioleoylphosphatidic acid (DOPA) and lysophospholipids showed an inhibitory effect on enzyme activity [11]. The molecular weight of the enzyme solubilized from porcine spleen microsomes was estimated to be 440 kd based on gel-filtration column chromatography, suggesting that this enzyme formed a complex with other protein molecules and membrane phospholipids, and that these phospholipids were necessary to maintain the enzyme activity [11].

Although PAF-CPT and the cholinephosphotransferase involved in phosphatidylcholine synthesis (PC-CPT) have several common features, however significant differences between the two enzymes concerning their behavior to detergents, DTT, ethanol, pH [8], as well as interactions with environmental membrane phospholipids containing phosphatidic acid (PA) and/or lysophospholipids [11] have been observed. All the above data support the hypothesis that PAF-CPT is a separate enzyme from PC-CPT, although further studies are required.

Investigation of PAF-CPT substrate specificity of several alkylacetylglycerol substrates has shown that the enzyme prefers alkyl substrates possessing either an acetyl or propionyl group at the *sn*-2 position. Also, an analog of alkylacetyl glycerol containing a methoxy substitution at the *sn*-2 position is not a substrate of PAF-CPT [8].

In the kidney, both *de novo* and remodeling biosynthetic routes can produce PAF either by intrinsic glomerular cells such as mesangial cells [16] or by infiltrating inflammatory cells. Apart from PAF physiological effects, its increased levels in kidney are involved in the pathogenesis and progression of renal damage [17–19]. The study of PAF metabolic enzymes in kidney, especially in mesangial cells, is of great importance since they regulate PAF levels both intracellularly and extracellularly.

In our previous studies, PAF-AH as well as remodeling and *de novo* acetyltransferases have been previously characterized in cortex and medulla from human kidney tissue [20–22], while remodeling PAF acetyltransferases have been characterized in human mesangial cells [23]. Although PAF metabolism has been described in mesangial cells [24, 25], as far as we know there are no direct studies on PAF-CPT in mesangial cells. The aim of the present work was a biochemical characterization of PAF-PCT in mesangial cells. Moreover, the effects of several bioactive compounds of Mediterranean diet and various drugs on PAF-CPT activity were tested in order to evaluate a possible beneficial effect of these factors on renal disorders.

## 2. MATERIALS AND METHODS

### 2.1. Materials and instrumentation

Centrifugations were performed in a Heraeus Labofug 400R and a Sorvall RC-5B refrigerated superspeed centrifuge (Sigma-Aldrich, St. Louis, Mo, USA) apart from the centrifugation at 100000×*g*, which was performed in a Heraeus-Christ, Omega 70000 ultracentrifuge (Hanau, Germany). Homogenizations were conducted at 40KHz with a VC50 supersonic sonicator of Sonics & Materials. (Sonics & Materials, Inc., Conn, USA). Aggregation studies were performed in a Chronolog aggregometer (model 400) (Havertown, Pa, USA) coupled to a Chrono-Log recorder (Havertown, Pa, USA) at 37°C with constant stirring at 1200rpm.

The electrospray ionization (ESI) mass spectrometry experiments were performed on an Electronspray MS-LCQ-Deca (Thermo Finnigan Ltd., Hertfordshire, UK), low flow, and mass spectrometer. Samples were dissolved in a small volume of HPLC grade methanol/water (70:30, v/v) 0.01 M in ammonium acetate. Electrospray samples are typically introduced into the mass analyzer at a rate of 3 *μ*L/minute. Nitrogen of purity 99.99% is used as nebulizing gas and as bath gas with flow rate of 3 *μ*L/minute. The spectrum analysis was contacted in the ion space of 50–1500 m/z and the conditions for the highest intensity of the summits with the lowest rupture were those that are shown below: spray voltage: 5 kV, capillary voltage: 7 V, capillary temperature: 275°C, lens-entrance voltage: −54 V, lens voltage: −22 V.

1-*O*-alkyl-2-*sn*-acetyl-glycerol (AAG) was purchased from BIOMOL International, L.P., Palatine House, Matford Court, Exeter, UK. CDP-choline, dithiothreitol (DTT), EDTA, MgCl_2_, analytical solvents, and Silica G for TLC method were purchased from Sigma Chemicals Co. and Merck KGaA Darmstadt Germany, respectively.

Bioactive lipid extracts of olive oil, olive pomace, sea bash, and sea bream were isolated as previously described [26, 27].

### 2.2. Cell culture

An established stable human mesangial cell line (HMC) was used in all the experiments (kindly donated by Dr Z. Varghese, Royal Free and University Collage Medical School, London, United Kingdom). HMCs were immortalized by transfection with T-SV40 and Hras oncogene, retaining many of the morphological and physiological features of normal human mesangial cells [28]. The cells were cultured as previously described [23] in medium containing RPMI 1640, 5% FCS, glutamine (2 mmol/L), penicillin (105 unit/L), streptomycin (0.1 g/L), amphotericin (2.5 × 10^−3^ g/L), insulin-transferrin (5 × 10^−3^ g/L), and sodium selenite (5 × 10^−6^ g/L).

### 2.3. Homogenization of mesangial cells and preparation of subcellular fractions

Homogenization of mesangial cells and preparation of subcellular fraction were carried out as previously described [23]. Briefly, mesangial cells were cultured in 75 cm^2^ flasks, the pellet of the cells was resuspended in homogenization buffer containing 0.25 M sucrose, 10 mM EDTA, 5 mM mercaptoethanol, 50 mM NaF, 50 mM Tris-HCl (pH 8), and was homogenized by sonication in −4°C. The homogenates were centrifuged at 500×*g* for 10 minutes to remove nucleus, whole cells, and debris. The pellets were discarded, a small portion of the supernatants was kept for protein determination and the rest of them were centrifuged at 20000×*g* for 20 minutes to remove mitochondria. Microsomes were isolated from cell homogenates after centrifugation of the final supernatant at 100000×*g* for 60 minutes. The resulting pellets were suspended in suspension buffer containing 0. 25 M sucrose, 1mM DTT, 50 mM Tris-HCl (pH 8), a small portion of the suspended microsomal pellet was kept for protein determination and the rest were aliquoted and stored at −20°C. All homogenization and fractionation procedures have taken place at −4°C.

### 2.4. DTT-insensitive cholinephosphotransferase (PAF-CPT) activity assays

DTT-insensitive CDP-choline:l-alkyl-2-acetyl-*sn*-glycerol cholinephosphotransferase (PAF-CPT) was assayed at 37°C for 20 minutes in a final volume of 200 *μ*L of reaction mixture containing 0.025–0.1 mg/mL of microsomal protein, 100 mM Tris-HCl (pH 8.0), 15 mM DTT, 0, 5 mM EDTA, 20 mM MgCl_2_, 1 mg/mL of BSA, 100 *μ*M of CDP-choline, and 100*μ* of AAG (added in the mixture in 2 *μ*L of ethanol). Variations in the incubation conditions, where needed, are noted in the figures and table legends.

The reactions were stopped by adding methanol (containing 2% acetic acid). The extraction and the purification of PAF were performed as previously described [21]. Briefly, the lipid products were extracted into chloroform according to the method of Bligh and Dyer [29], while the separation of the lipid extracts was achieved by a TLC method on Silica Gel G in chloroform:methanol:acetic acid:water (100:57:16:8, v/v). Lipid fractions were visualized by I_2_ exposure and phospholipid products were identified by cochromatography with known standards. PAF fractions were scrapped off and extracted with chloroform according to the method of Bligh and Dyer [29].

The PAF solutions in chloroform were evaporated under a stream of nitrogen, redissolved in BSA (0.25% w/v in saline), and the produced PAF levels were estimated by washed rabbit platelet aggregation [1]. This procedure was used for all the quantitive determinations mentioned in results. The TLC fraction with PAF-like activity was subjected to ESMS analysis.

In order to study the in vitro effect of drugs and food extracts on PAF-CPT activity, these factors were dissolved in 40 mg/mL of BSA and then added on the reaction mixture. PAF-CPT enzymatic assay was performed in the presence of several concentrations of each factor in the reaction mixture (while BSA concentration in all cases was 1 mg/mL).

### 2.5. Analytical methods

Protein concentrations, determined according to Lowry et al. [30], were based on bovine serum albumin as the protein standard.

### 2.6. Recovery of the method

In order to evaluate the percentage of the recovery of the product (PAF) of the enzymatic reaction from all the above steps and procedures of this method, the microsomal fraction was heated at 100°C for 20 minutes and the inactive microsomal protein was added in the above-mentioned reaction mixture that also contains specific concentration of standard PAF. This experiment was performed six times. Estimation of PAF levels from these experiments was carried out as mentioned above. The percentage of the recovery of the product (PAF) of the enzymatic reaction from all the above steps and procedures of this method has a mean value of 62.20 ± 5.32%. This value was taken into account in the calculation of all PAF-CPT activities.

### 2.7. Statistical analysis

Data are expressed as mean values ± SD using Microsoft Excel. The linear or nonlinear regressions of enzymes kinetics were also made using Microsoft Excel.

## 3. RESULTS

### 3.1. PAF-CPT activity of HMC

PAF-CPT activity was determined by produced PAF levels that were separated by TLC. The TLC fraction with PAF-like activity gave identical ESMS fragments to the ones of synthetic PAF (16:0), M.W.: 524 (data not shown).

The specific activity of PAF-CPT of HMC was detected mainly in the microsomal fraction (100 000×*g* pellet) with a value of 0.36 ± 0.20nmol/mg/min. It was also detected in the homogenate fractions (500×*g*), and in the mitochondrial fractions (20000×*g*), in a lower range. PAF-CPT activity was not detected in the cytoplasmic fractions (100 000×*g* supernatants). The results are shown in Table 1. All subsequent experiments were performed with microsomal preparations. PAF-CPT specific activity was stable for a period of 30 days and was reduced at 50% after 45-day storage at − 20°C, respectively.

### 3.2. Effect of temperature and pH on PAF-CPT activity of HMC

Experiments were carried out in order to found the optimum conditions for the action of the enzyme. The temperature activity profiles were bell-shaped showing an optimum at 37°C. Heating of samples at 50°C and 60°C for 20 minutes resulted in 80% and 100% inactivation of the existing enzyme activity, respectively.

**Figure 1:**
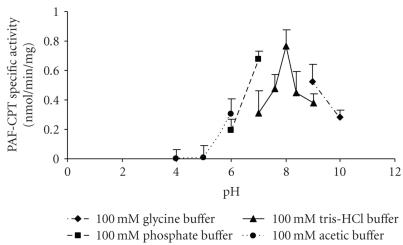
Effect of pH on PAF-CPT specific activity of mesangial cells: microsomal fractions of HMC 0.05 mg/mL were incubated in the presence of different buffer solutions. Results are the average of two independent determinations using different enzyme preparations performing duplicate samples.

In order to investigate the dependence of PAF-CPT activity on pH, four different buffer solutions of various pH values were utilized, namely, 100 mM acetic (CH_3_COOH–CH_3_COONa) buffer pH 4–6, 100 mM phosphate (NaH_2_PO_4_–Na_2_HPO_4_) buffer pH 6–7, 100 mM Tris-HCl buffer pH 7–9, and 100mM Glycine buffer pH 9–10. The pH activity profile was bell-shaped showing an optimum pH range at 8. The results are shown in Figure 1.

### 3.3. Effect of BSA on PAF-CPT activity of HMC

**Figure 2:**
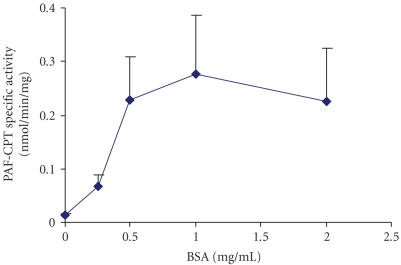
Effect of BSA concentration on PAF-CPT specific activity of mesangial cells: microsomal fractions of HMC 0.05 mg/mL were incubated in the presence of different concentrations of BSA. Results are the average of two independent determinations using different enzyme preparations performing duplicate samples.

Microsomal fractions of HMC 0.05 mg/mL were incubated in the presence of different concentrations of BSA in a range of 0.1–2 mg/mL in the reaction medium. As shown in Figure 2, PAF synthesis is increased (*P* < .05) at BSA concentrations ranging from 0.5 to 2.

### 3.4. Effect of dithiothreitol (DTT) on PAF-CPT activity of HMC

**Figure 3:**
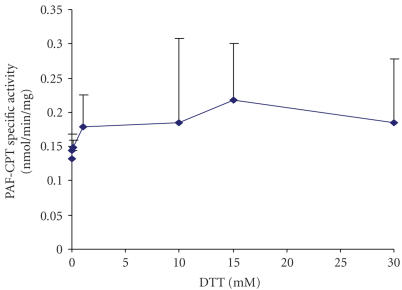
Effect of DTT concentration on PAF-CPT specific activity of mesangial cells: microsomal fractions of HMC 0.05 mg/mL were incubated in the presence of different concentrations of DTT. Results are the average of two independent determinations using different enzyme preparations performing duplicate samples.

Microsomal fractions of HMC 0.05 mg/mL were incubated in the presence of different concentrations of DTT in a range of 0.01–20 mM in the reaction medium. As shown in Figure 3, PAF synthesis is slightly increased (*P* > .05) at DTT concentrations above 1mM; it exhibits optimal stimulation at 15 mM DTT.

### 3.5. Effect of divalent cations and EDTA and several chemicals on PAF-CPT activity of HMC

Microsomal fractions of HMC 0.05 mg/mL were incubated in the presence of different concentrations of both cations in the absence or in the presence of the chelate reagent EDTA. The results are summarized in Tables 2 and 3. The highest activity was obtained with 20 mM Mg^2+^ in the presence of 0.5 mM EDTA (*P* < .05), which was routinely used. In contrast, Ca^2+^ levels of 0.1mM caused inhibition of PAF-CPT in the absence of EDTA (*P* < .05), which was partially reduced by the presence of the chelate reagent.

**Figure 4:**
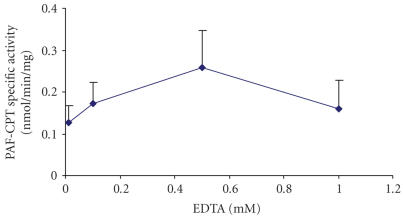
Effect of the concentration of the chelate reagent EDTA in the presence of 20 mM Mg^2+^ on PAF-CPT specific activity of mesangial cells: microsomal fractions of HMC 0.05 mg/mL were incubated in the presence of different concentrations of EDTA. Results are the average of two independent determinations using different enzyme preparations performing duplicate samples.

In order to study the effect of EDTA concentration on the PAF-CPT activity of HMC, microsomal fractions of HMC 0.05 mg/mL were incubated in the presence of different concentrations of EDTA in a range of 0.01–10 mM, as well as in the presence of 20 mM Mg^2+^, in the reaction medium. As shown in Figure 4, the maximum enzyme activity occurred at 0.5 mM EDTA final concentration in reaction medium, which was routinely used. Concentration of EDTA of 10 mM abolished PAF-CPT activity.

In conclusion, the presence of 1 mg/mL BSA, 15mM DTT, 20 mM Mg^2+^, and 0.5 mM EDTA in the reaction medium of PAF-CPT assay resulted in maximum activity, and was routinely used.

### 3.6. Effect of chemicals (NaF and Pefabloc) on PAF-CPT activity of HMC

The presence of 50 mM of NaF, a protease inhibitor, in the reaction medium reduced PAF-CPT activity up to 40%, while the presence of 0.1mM of Pefabloc, a sulfonyl-type serine protease inhibitor, had no significant effect on the enzyme action suggesting the absence of serine(s) in the active site of the enzyme.

### 3.7. Dependence of PAF formation by protein concentration and incubation time

**Figure 5:**
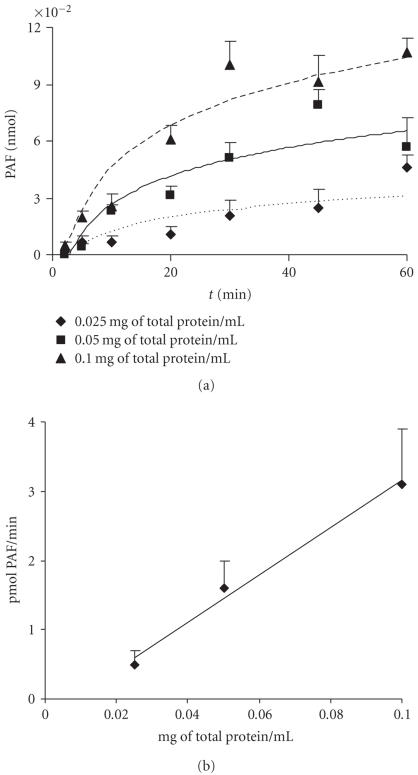
Dependence of PAF formation on incubation time and protein concentration: (a) time course of PAF production using 0.025, 0.05, and 0.1 mg total protein/mL; (b) CPT activity as a function of protein concentration at a fixed incubation time of 20 minutes. Experiments were performed with microsomal fractions of HMC in the presence of 100 *μ*M AAG and 100 *μ*M CDP-choline. Results are the average of two independent determinations using different enzyme preparations performing duplicate samples.

The kinetics of PAF formation in relation to time and protein concentration is shown in Figure 5. The total amount of PAF formed at the end of each incubation time decreased as protein concentration decreased (Figure 5(a)). A linear relationship between the initial velocity and total microsomal protein up to 0.1 mg/mL was found for 20 minutes incubation time (Figure 5(b)). In order to achieve the maximum yield of reaction, 0.05 mg/mL protein and 20-minute incubation time were routinely used.

### 3.8. Effect of substrates concentration on PAF-CPT activity and kinetic parameters

**Figure 6:**
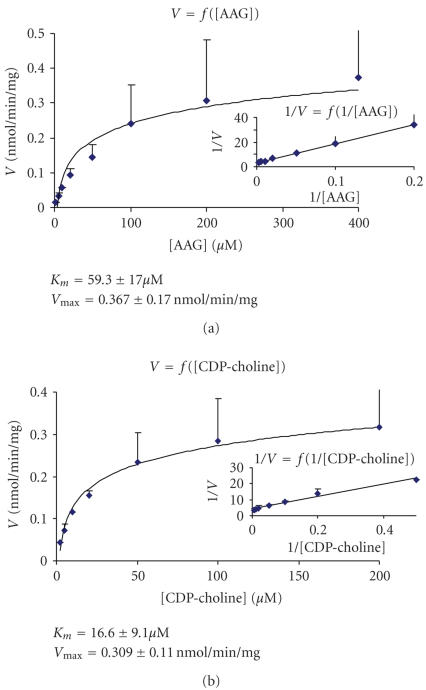
Effect of substrate concentration on PAF-CPT activity: (a) activity of PAF-CPT as a function of AAG at fixed concentration of CDP-choline (100 *μ*M) and the relative Lineweaver-Burk plot; (b) activity of PAF-CPT as a function of CDP-choline at fixed concentration of AAG (100 *μ*M) and the relative Lineweaver-Burk plot. Experiments were performed at microsomal fractions using 0.05 mg/mL protein in optimum conditions. Results represent the average ± SD of three independent determinations using different enzyme preparations performing duplicate samples.

The activity of PAF-CPT was determined at different AAG concentrations ranging from 2 to 400 *μ*M at a fixed concentration of CDP-choline 100 *μ*M (Figure 6(a)) and at different CDP-choline concentrations ranging from 2 to 200 *μ*M at a fixed concentration of AAG 100 *μ*M (Figure 6(b)). The results revealed that enzyme exhibited classical Michaelis-Menten kinetics with respect to AAG as well as to CDP-choline. The kinetic parameters of the enzyme showed on Figure 6 were calculated using Lineweaver-Burk plot.

### 3.9. Effect of various drugs and lipid extracts

In order to examine the in vitro effect on PAF-CPT activity of various drugs and lipid extracts of nutrients of Mediterranean diet, preliminary experiments were conducted. Microsomal fractions were isolated from HMC and the enzymatic assay of PAF-CPT was performed in the presence of several concentrations of each compound in the reaction mixture. The concentration of each compound that inhibited the maximum inhibitory effect against PAF-CPT activity is represented on Table 4. Drug trade names and food origin of the bioactive compounds, as well as their reported biological actions, are also shown on Table 4.

## 4. DISCUSSION

In this study, the presence of PAF-CPT activity in HMC was detected for the first time. Moreover, the characterization of PAF-CPT of HMC was also performed for the first time. In order to evaluate PAF-CPT activity of HMC, a new enzymatic assay procedure was achieved. In contrast with all previous reported enzymatic assays for the determination of PAF-CPT, (a) in this assay the amount of the added sample protein in the reaction mixture is much lower, giving the opportunity to detect PAF-CPT activity in samples with low protein distribution, (b) this assay does not need the use of radiolabeled compounds, reducing though the risk and expense of the procedure.

According to this assay, PAF-CPT activity was detected mainly in the microsomal fraction and its biochemical characteristics were found similar to previously published data from other groups in other types of cells and tissues [7–12, 14, 15]. Specifically, PAF-CPT activity is significantly increased in the presence of specific concentrations of BSA, DTT, Mg^2+^, and EDTA.

DTT was added in reaction mixture in order to inhibit PC-CPT, as posphatidylcholine (PC) synthesis by its cholinephosphotransferase, PC-CPT, is known to be strongly inhibited at these DTT concentrations [8, 14]. Moreover, DTT seems to increase PAF-CPT activity.

In accordance to reported data, exogenous Mg^2+^ are essential for enzyme action, while exogenous Ca^2+^ exhibit a dose-dependent inhibitory effect that was partly abolished by the presence of EDTA. In several studies, various chelate reagents were used in order to reduce the inhibitory effect of Ca^2+^ on PAF-CPT [8–12, 14]. It is well known that the chelate reagent EDTA prefers to bind to Ca^2+^ than to Mg^2+^ in the copresence of these divalent cations in the reaction medium.

In the absence of exogenous Ca^2+^, a small range of EDTA concentration, near 0.5 mM, enhances enzyme activity by primary binding endogenous Ca^2+^ than Mg^2+^. PAF-CPT of HMC required a specific concentration of EDTA for its activation as both lower and higher concentrations result in its inactivation, by either the inhibitory effect of the unbounded Ca^2+^ or by the reduction of Mg^2+^ levels from its binding with the EDTA excess, respectively. However, in these high concentrations of EDTA (up to 10 mM), it is possible that the inactivation of the enzyme may be caused not only by the binding of Mg^2+^, but also by the effect of this chelate reagent directly on the enzyme.

In addition, PAF-CPT exhibited classical Michaelis-Menten kinetics with respect to both its substrates, AAG and CDP-choline. The kinetic parameters of the enzyme (*K*_*m*_ and *V*_*max*_) for both substrates were similar to reported ones in other tissues and cells [7, 8, 14, 15].

On the other hand, pefabloc (a sulfonyl-type serine protease inhibitor) had no significant effect on the enzyme action supporting the absence of serine(s) in the active site of the enzyme. Moreover, since Pefabloc is also PAF AH inhibitor [42], the formed PAF is not affected from the possible presence of PAF-AH.

It has been reported that several other factors can regulate PAF-CPT, suggesting that this enzyme serves as an important control point in the de novo synthesis of PAF [7–12, 14]. For example, PAF-CPT forms a complex with membrane phospholipids and other protein molecules, while environmental membrane phospholipids seem to regulate this enzyme [11].

Moreover, the neurotransmitters acetylcholine and dopamine as well as the activators of protein kinase C 12-myristate-13-acetate phorbol (PMA) and oleoylacetylglycerol (OAG) (molecules that are implicated in several pathological situations) also stimulate the de novo PAF-CPT, while they do not activate the enzymes of the remodelling pathway [8, 9, 43,44].

Although it is believed that the remodeling pathway is activated under inflammatory situations, the de novo pathway, and especially PAF-CPT, may contribute to systemic disorders such as cancer [13] and central nervous system failure [12], by a slightly long-term enhance of PAF-CPT activity that could lead to steady increased levels of PAF, which subsequently maybe implicated in systemic disorders. These pathological situations may be reversed by inhibiting PAF-CPT, as it was reported in two studies where the reduction of PAF-CPT activity in human renal cell carcinoma (RCC) in patients who had received IFNa, as well as in brain striatum of rats who had received CDP-choline, resulted in reduced levels of PAF with beneficial effects (inhibition of tumor progression and various disorders of the central nervous system) in both cases [12, 13].

In this study, preliminary in vitro experiments were conducted in order to test the effects of several bioactive compounds of Mediterranean diet and various drugs, which are related to several manifestations where inflammation dominates [26, 27, 31–41, 45, 46], on PAF-CPT activity. The results have revealed that the drugs Symvastatin and Rupatadine, and several bioactive compounds of Mediterranean diet, such as resveratrol (found also in wine) and polar lipids of olive oil, olive pomace, and sea bass, exhibit an inhibitory effect on PAF-CPT of HMC in a dose-dependent manner. Several other drugs such as INFa, Aspirin, and Tinzaparin, as also as other bioactive compounds of Mediterranean diet such as polar lipids of see bream inhibited PAF-CPT in a non-dose-dependent manner.

In conclusion, these data demonstrate that DTT-insensitive cholinephosphotransferase (PAF-CPT) activity is present in human mesangial cells and the biochemical properties and kinetic parameters of this enzyme of HMC were established for the first time. Concerning previous data, PAF-CPT of mesangial cells seems to have similar properties with PAF-CPT characterized in several cells and tissues including human kidney. PAF production in kidney, mainly by mesangial cells, is involved in the pathogenesis of renal damage. The characterization of PAF-CPT activity in human mesangial cells enables further investigation of PAF-CPT regulatory mechanisms, and therefore its contribution in PAF production under physiological and pathological conditions. Several drugs and bioactive compounds of Mediterranean diet with beneficial effects in various pathological conditions [26, 27, 31–41] seem to exhibit an inhibitory effect on PAF-CPT activity that maybe correlate with their general biological action. It is possible that the inhibition of PAF-CPT activity in several pathological manifestations (mainly systemic) can restore pathologically long-term slightly increased basal PAF levels to their physiological ones, and therefore might reverse these pathological conditions. This new point of view need to be further studied.

## Figures and Tables

**Table 1:** Subcellular specific activity of PAF-CPT from human mesangial cell preparations (*n* = 3).

Subcellular fractions	PAF-CPT specific activity
	(nmol/min/mg)

Homogenate fractions	0.10 ± 0.05
(500×*g* supernatant)	
Mitochondrial fractions	0.02 ± 0.01
(20000×*g* pellet)	
Cytoplasmic fractions	Nondetected
(100000×*g* supernatant)	
Microsomal fractions	0.36 ± 0.20
(100000×*g* pellet)	

**Table 2:** Effect of Mg^2+^ cation on PAF-CPT activity of HMC. Results represent the average of two independent experiments using different enzyme preparations performing duplicate samples.

Mg^2+^ cation	Concentration in assay (mM)	PAF-CPT specific activity (nmol/min/mg)

Exogenous Mg^2+^ ([EDTA] = 0)	0	Not detected
Exogenous Mg^2+^ ([EDTA] = 0)	0.1	0.0003
Exogenous Mg^2+^ ([EDTA] = 0)	1	0.0012
Exogenous Mg^2+^ ([EDTA] = 0)	10	0.0036
Exogenous Mg^2+^ ([EDTA] = 0)	20	0.067
Exogenous Mg^2+^ ([EDTA] = 0.5 mM)	20	0.25

**Table 3:** Effect of Ca^2+^ cation on PAF-CPT activity of HMC. Results represent the average of two independent experiments using different enzyme preparations performing duplicate samples.

Ca^2+^ cation	Concentration in assay (mM)	PAF-CPT activity (%)

Exogenous Ca^2+^ ([EDTA] = 0.5 mM, [Mg^2+^] = 20 mM)	0	100
Exogenous Ca^2+^ ([EDTA] = 0, [Mg^2+^] = 20 mM)	0	67.4
Exogenous Ca^2+^ ([EDTA] = 0, [Mg^2+^] = 20 mM)	0.1	14.1
Exogenous Ca^2+^ ([EDTA] = 0.5 mM, [Mg^2+^] = 20 mM)	0.1	33.3
		
Exogenous Ca^2+^ ([EDTA] = 0, [Mg^2+^] = 20 mM)	1	1.41
Exogenous Ca^2+^ ([EDTA] = 0.5 mM, [Mg^2+^]= 20 mM)	1	3.70

**Table 4:** In vitro effects of various drugs and bioactive compounds of Mediterranean diet on PAF-CPT activity of HMC. Preliminary results represent the average of two independent experiments using different enzyme preparations performing duplicate samples. Results are expressed as percentage of inhibition of PAF-CPT activity versus control (absence of these factors).

Bioactive compound	Drug name/food origin	Action	Concentration in assay	Inhibition of PAF-CPT (%)

Rupatadine	Rupafin	(i) Antiallergic [31]	20 ng/*μ*L	60
		(ii) PAF-antagonist [31]		
Simvastatin	Zocor	(i) Antiatherogenic [32]	40 ng/*μ*L	92
		(ii) Anticancer [33]		
INFa	IntronA	Anticancer [34]	250 IU/*μ*L	51
Tinzaparin	Innohep	(i) Antithrombotic [35]	0.25 IU/*μ*L	30
		(ii) Anticancer [36]		
Salicylic acid	Aspirin	(i) Anti-inflammatory [37]	250 ng/*μ*L	38
		(ii) Anticancer [38]		
Resveratrol	—	(i) Antioxidant [39]	100 *μ*M	39
		(ii) Anti-PAF [39]		
		(iii) Anticancer [39]		
Polar lipids	Olive oil	(i) Antiatherogenic [40]	5.2 ng/*μ*L	33
		(ii) Anti-PAF [40]		
		(iii) Anticancer [41]		
Polar lipids	Olive pomace	(i) Antiatherogenic [26, 40]	1.8 ng/*μ*L	48
		(ii) Anti-PAF [26, 40]		
Polar lipids	Sea bass	Anti-PAF [27]	59 ng/*μ*L	45
Polar lipids	Sea bream	Anti-PAF [27]	123 ng/*μ*L	17
